# Clinical efficacy of adjuvant chemotherapy in the treatment of pT4 stage II colorectal cancer with defective mismatch repair status: A protocol for systematic review and meta-analysis

**DOI:** 10.1097/MD.0000000000020693

**Published:** 2020-06-26

**Authors:** Li-Bin Huang, Ting-Han Yang, Lie Yang, Yong-Yang Yu, Zi-Qiang Wang, Cun Wang, Zong-Guang Zhou

**Affiliations:** Department of Gastrointestinal Surgery, West China Hospital and State Key Laboratory of Biotherapy, Sichuan University, Chengdu, China.

**Keywords:** adjuvant chemotherapy, defective mismatch repair, high-risk factor, meta-analysis, protocol, stage II colorectal cancer

## Abstract

**Background:**

The aim of this systematic review and meta-analysis is to assess the efficacy of adjuvant chemotherapy in patients with stage IIB/C CRC and defective mismatch repair (dMMr) status, and to evaluate what is the determinant risk factor for adjuvant chemotherapy in those patients.

**Method:**

A systematic search of PubMed, EMBASE, Web of science, Cochrane Library databases will be performed. All RCTs published in electronic databases from inception to March 19, 2020, with language restricted in English will be included in this review study. Two reviewers will independently perform the Study selection, data extraction, quality assessment, and assessment of risk bias and will be supervised by third party. Outcomes consisted of overall survival, progression-free survival and sufficient information to extract hazard ratios and their 95% confidence intervals and it will be calculated to present the prognostic role of adjuvant chemotherapy in patients with stage IIB/C CRC and dMMR status using Review Manager version 5.3 when there is sufficient available data.

**Results:**

The results of this systematic review and meta-analysis will be submitted to a peer-reviewed journal for publication.

**Conclusion:**

This study will summarize up-to-date evidence to assess the efficacy of adjuvant chemotherapy in patients with stage IIB/C CRC and dMMR status and provide a scientific and practical suggestions for treatment decision-making.

**Registration:**

This protocol has been registered on the International Platform of Registered Systematic Review and Meta-Analysis Protocols (INPLASY) with a registration number of INPLASY202050019.

## 1 Introduction

Stage IIB/C (T4a-bN0) colorectal cancer (CRC) have been demonstrated to have a worse oncological outcome compared with stage IIIA disease.^[1,2]^ This phenomenon can be explained by several factors, including insufficient lymph node harvest, inherent high-risk biology of T4 tumors and the lack of adjuvant chemotherapy(ACT) for Stage IIB/C disease.^[3]^ Despite the adverse impact of T4 tumors on prognosis, whether patients with T4 tumors could benefit from ACT is still under debate. An early pooled analysis showed that patients with high-grade T4 tumors did not have a better outcome if they received postoperative fluorouracil-based chemotherapy when compared with surgery alone (5-year survival 72% vs 69%).^[4]^ In contrast, in an analysis of 1697 patients with stage II CRC, survival benefits of ACT were observed only in patients with T4 lesions but not other risk factors such as insufficient nodal sampling (<12 lymph nodes), presence of lymphovascular or perineural invasion, and poor differentiation.^[5]^ A later Dutch study enrolled 4940 patients with at least 1 high factor further demonstrated that ACT was associated with higher 3-year overall survival in T4 disease only (hazard ratios (HR) = 0.43, 95% confidence interval (CI) = 0.28–0.66). A recent systematic review and meta-analysis, which included 23 cohort studies and 1 randomized controlled trial, demonstrated that ACT improves overall survival in patients with T4 tumors (HR = 0.47, 95% CI = 0.38–0.59) but has no effect on disease-free survival in this group.^[6]^ In spite of the lack of data from randomized controlled trials to support the use of ACT in patients with T4 tumors, the American Society of Clinical Oncology(ASCO) guideline and the National Comprehensive Cancer Network (NCCN), suggested that T4 tumors should be taken into account about the potential benefits of ACT. Along with clinicopathologic features, molecular factors associated with prognosis and response to ACT play a role in stage II CRC, especially the mismatch repair status (MMR). Defective mismatch repair (dMMR) is more prevalent in stage II when compared to stage III CRC (21%–22% vs 12%–14%).^[7,8]^ Currently, evidence have shown that dMMR or MSI-H tumor status is a prognostic marker of a better outcome.^[9–11]^ In addition, dMMR status may also be a predictive marker of decreased benefit and even a harmful influence from ACT in patients with stage II CRC.^[10–12]^ Therefore, guidelines recommended that adjuvant chemotherapy should not be given to patient with stage II disease without high-risk features. For Stage IIB/C disease, the pT4 itself is a high-risk factor, but it remains unclear whether patients with T4 tumors and dMMR should receive ACT. The algorithm of the European Society for Medical Oncology (ESMO) guideline indicates that patients with T4 tumor should consider ACT regardless of MMR status,^[13]^ whereas the NCCN guidelines suggest that patients with pT3-4, N0, M0 (MSI-H/dMMR) disease could have surgery alone.^[14]^ This difference between the guidelines introduce a dilemma in decision making for adjuvant chemotherapy in Stage IIB/C CRC. The purpose of this systematic review and meta-analysis is to evaluate what is the determinant factor for ACT in patients with T4 tumors and dMMR status.

## 2 Method and design

The protocol of this systematic review and meta-analysis has been registered on the International Platform of Registered Systematic Review and Meta-Analysis Protocols (INPLASY) with a registration number of INPLASY202050019. The registered website for this protocol is https://inplasy.com/inplasy-2020-5-0019/. We will develop the protocol strictly according to the guidelines of Preferred Reporting Items for Systematic Reviews and Meta-Analyses protocols (PRISMA-P).

### 2.1 Literature search strategy

A systematic search of PubMed, EMBASE, Web of science, Cochrane Library databases will be performed. And the included references, academic conferences, and network resources in the literature were inquired at the same time to find out the research that may meet the inclusion criteria. All Randomized controlled trials (RCT) published in electronic databases from inception to March 19, 2020, with language restricted in English will be included in this review study. We will manage all references and duplicates using EndNote X9 citation management software. The clinical problems were refined by the principle patient, intervention, contrast, outcome, study (PICOS)

P: pT4 stage II colorectal cancer patients with defective mismatch repair status;

I: adjuvant chemotherapy;

C: adjuvant chemotherapy vs observation;

O: prognostic effectiveness;

S: RCTs.

The Medical Subject Headings (MeSH), text words, and word variants for “T4N0M0”, “pT4”, “T4”, “stage 2”, “stage II”, “colorectal cancer”, “colorectal neoplasms”, “dMMR”, “MSI-H”, “dMMR/MSI-H”, “defective mismatch repair”, “high-risk”, “adjuvant chemotherapy”, “post-operative chemotherapy”, “prognostic”, “prognosis”, “overall survival,”“OS,” “progression free survival,” and “PFS” are used and combined in the searches. This search strategy will be modified to be suitable for other electronic databases.

### 2.2 Inclusion and exclusion criteria

#### 2.2.1 Type of study

Randomized controlled trial of ACT in the treatment of dMMR pT4 stage II CRC. Whether or not the blind method and distribution concealment are mentioned.

#### 2.2.2 Types of participants

It included patients who underwent radical resection and dMMR pT4 stage II CRC was confirmed by pathologic or histologic examination after surgery. The tumor that yielded negative staining results for at least one of the MMR proteins, MLH1, MSH2, MSH6, PSM6 were classified as dMMR tumors, and all others were classified as pMMR tumors.

#### 2.2.3 Types of interventions

Receiving adjuvant chemotherapy versus observation. There will be no restrictions on the type, dose, frequency of ACT. The control group (observation group) will not receive any type of ACT. Studies to compare the effect of different ACT strategies without only observation group will be excluded.

#### 2.2.4 Outcome measurements

The primary outcomes consisted of overall survival (OS), progression-free survival (PFS) and sufficient information to extract HRs and their 95% CIs. Secondary outcomes consisted of other clinical and pathological high-risk factors, included intestinal perforation, intestinal obstruction, fewer than 12 sample of lymph nodes, lymphovascular invasion (LVI), perineural invasion (PNI), poor differentiated histology, and close or indeterminate or positive margins.

### 2.3 Data extraction

#### 2.3.1 studies selection

**Figure 1:**
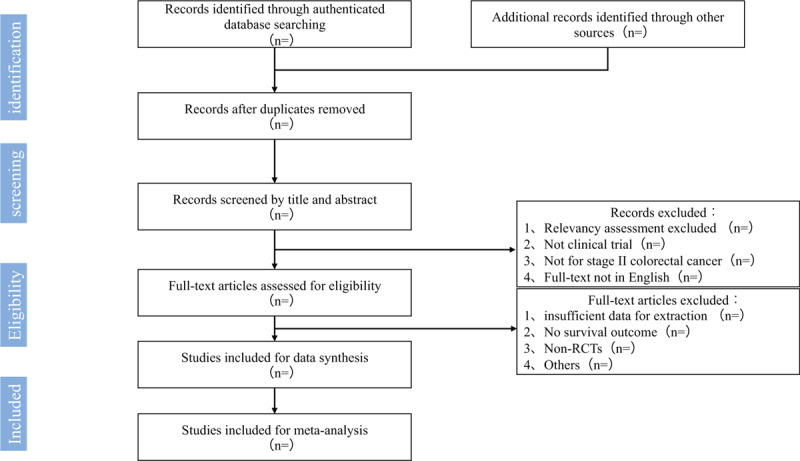
Flow diagram of studies identified.

All of the searched studies will be extracted by 2 authors (LBH and THY) independently. The duplicated studies will be removed by using the function of citation management software. Firstly, the title and abstract of initial searched studies will be scanned, case reports, letters, conference summaries, and the studies not meet the inclusion criteria will be excluded. All excluded studies should record the reasons for the exclusion. Secondly, the full text will be further assessed with the inclusion criteria. Documents with incomplete information or missing data can be obtained by contacting the original corresponding author through e-mail, If the missing data cannot be obtained, it will be excluded from analysis. Thirdly, the results of included studies will be cross-checked by 2 authors, if there were difference, the third-party (CW) will be consulted. The studies selection procedures are shown in Preferred Reporting Item for Systematic review and Meta- analysis protocol (PRISMA-P) flow chart (Fig. 1).

#### 2.3.2 Data extraction

The data extraction will include

1. basic information of the articles: title, name of first author, published year, name of published magazine, country, study design, sample size, blind method, randomization;
2. participants characteristics: sample size, mean age, proportion of sex, pathological or histologic results, intervention (including the strategies of ACT);
3. prognosis effective outcome data: primary outcome measures, secondary outcome measures, follow-up time, adjusted HRs and 95% CIs;
4. study conclusions. After data extraction 2 authors will cross-check the results, disagreement which existed between 2 author's results will be solved by discussion and the extraction data will be checked by CW.

### 2.4 Quality assessment

The quality of evidence of outcomes will be assessed by two authors(LBH and THY) according to the “Bias Risk Assessment” tool recommended by Cochrane Collaboration Network (Version 5.1.0) which include selection bias (method of randomization and allocation concealment), information bias (masking of outcome adjudicators), and bias in the analysis (intention to treat analysis and completeness of follow-up). The strength of the body of evidence will be graded into 3 levels, “High Risk”, “Low Risk”, “Unclear”. Disagreement which existed between 2 author's results will be solved by discussion and settled through consultation with the third party (CW). Bias risk assess figure will be drawn by RevMan software 5.3.

### 2.5 Data Statistical analysis

We will employ the RevMan software 5.3 software to evaluate the correlations between intervention and OS and DFS using HRs and 95% CIs. If HRs and 95% CIs cannot be obtained from the original study, we will figure out these values using the methods reported by Parmar et al^[15]^ and Tierney et al^[16]^ The heterogeneity will be analyzed before meta-analysis, we will use *I*^*2*^ statistics to assess heterogeneity across included studies. If *P-*value <.10 and/or *I*^*2*^ < 50%, it indicate that the heterogeneity among included studies were small we will pool data across studies using fixed-effects model for meta-analysis. If *I*^*2*^ >50%, we will use random-effects model to make meta-analysis, and sensitivity analysis or subgroup analysis is needed to identify the sources of heterogeneity among the included studies. And the 2-side *P* value < .05 in Z-test will be considered as statistically significant.

### 2.6 Sensitivity analysis

In order to ensure the stability of primary outcome, we will perform sensitivity analysis by excluded those studies with high risk of bias according to the sample size, study design, heterogeneity qualities, and statistical model (random-effects or fixed-effects model) and with non-informative prior distributions for the heterogeneity parameters. If result of sensitivity analysis is quite different from meta-analysis, it should be considered to make a descriptive analysis.

### 2.7 Subgroup analysis

We will perform subgroup analysis to find out heterogeneity parameters. Subgroup analysis will be done based on sex, age, intestinal perforation, intestinal obstruction, fewer than 12 sample of lymph nodes, lymphovascular invasion (LVI), perineural invasion (PNI), poor differentiated histology, and close or indeterminate or positive margins.

### 2.8 Publication bias

Egger test and funnel plot will be performed to assess the publication bias when applicable.

### 2.9 Ethical approval and dissemination

The ethical approval of clinical research is not suitable for this study.

## 3 Discussion

Stage II CRC accounts for 25% of all cases of CRC. In the past 2 decades, CRC screening have drawn wide attention and more early stage CRC are detected, as the incidence rate of stage II CRC has increased rapidly.^[17]^ Patients with stage II CRC have relatively good outcomes, but there are still 20% to 25% develop recurrence and/or metastasis.^[1,2]^ Previous evidence indicate that survival benefit has not been demonstrated for the addition of oxaliplatin to 5-FU/leucovorin in stage II colon cancer.^[18,19]^ Recent studies showed FOLFOX is reasonable for stage II patients with multiple high-risk factors and it is not indicated for low-risk patients with stage II CRC.^[20]^

At present, high-risk and low-risk factors recommended in clinical guidelines from major groups are controversial. The NCCN guideline reported that the prognosis of patients with dMMR in stage II CRC was good and they did not benefit from 5-FU/leucovorin-based ACT even when they have other high-risk factors such as pT4 pathologic tumor stage.^[14]^ But in the ESMO clinical treatment recommendations indicated that all patients with pT4 stage II CRC should receive ACT.^[13]^ Although these recommendations were based on high level clinical evidence, whether these patients with both high-risk factor (pT4) and low-risk factor (dMMR) could benefit from ACT remains unclear. Therefore, there appears to be a great deal of interstudy heterogeneity in current reports, a large-cohort retrospective analysis showed there was no significant association between survival and MMR status or clinical risk factors. Results from QUASAR study found that dMMR was prognostic but it did not predict response to ACT.^[21]^ In contrast, the analysis from National Cancer Database found that ACT was associated with improved survival (HR, 0.76; *P* < .001),^[22]^ and other results from Netherlands showed that this benefit of ACT in patients with stage II CRC may be only limited to those with pT4 lesions.^[23]^ It is still unclear that how the risk-factors affect to prognosis, and many patients without high-risk factors still have a recurrence.^[24]^ Furthermore, there are no clear predictive factors of benefit from ACT in patients with stage II CRC.^[25]^ Regarding the aforementioned limitations, the robustness and reliability of the results are affected. The efficacy of ACT in patient with stage II colorectal cancer remains uncertain, Therefore, the generalizable and sufficient data sets are essential to provide a systematic estimation in dMMR patients with pT4 stage II CRC. We will perform a systematic review to provide the most comprehensive evaluation of effectiveness of ACT in those patients by including currently available data from RCTs. This study will provide a scientific and practical conclusion through a systematic review and meta-analysis for treatment decision-making guidance the use of adjuvant therapy for patients with stage II CRC.

## Author contributions

**Conceptualization:** Ting-Han Yang, Lie Yang, Zi-Qiang Wang, Zong-Guang Zhou.

**Data curation:** Li-Bin Huang, Ting-Han Yang, Lie Yang, Yong-Yang Yu.

**Formal analysis:** Li-Bin Huang, Zong-Guang Zhou.

**Funding acquisition:** Ting-Han Yang, Zong-Guang Zhou.

**Investigation:** Li-Bin Huang, Ting-Han Yang.

**Methodology:** Li-Bin Huang, Ting-Han Yang, Lie Yang.

**Project administration:** Lie Yang, Zi-Qiang Wang, Zong-Guang Zhou.

**Resources:** Yong-Yang Yu, Zi-Qiang Wang.

**Supervision:** Lie Yang, Yong-Yang Yu, Zi-Qiang Wang, Zong-Guang Zhou.

**Validation:** Li-Bin Huang, Yong-Yang Yu, Zong-Guang Zhou.

**Writing – original draft:** Li-Bin Huang, Ting-Han Yang.

**Writing – review & editing:** Lie Yang, Yong-Yang Yu, Zi-Qiang Wang, Zong-Guang Zhou.

LBH, THY, CW planned and designed the research. LBH, THY, LY, YYY, CW tested the feasibility of the study. YYY, LY CW, ZQW, ZGZ provided methodological advice, LY, CW, ZQW, ZGZ polished and revised the manuscript. LBH, THY, CW wrote the manuscript. All authors approved the final version of the manuscript.
